# Estimation of contrast agent bolus arrival delays for improved reproducibility of liver DCE MRI

**DOI:** 10.1088/0031-9155/61/19/6905

**Published:** 2016-09-12

**Authors:** Manil D Chouhan, Alan Bainbridge, David Atkinson, Shonit Punwani, Rajeshwar P Mookerjee, Mark F Lythgoe, Stuart A Taylor

**Affiliations:** 1 University College London (UCL) Centre for Medical Imaging, Division of Medicine, UCL, London, UK; 2 Department of Medical Physics, University College London Hospitals NHS Trust, London, UK; 3 University College London (UCL) Institute for Liver and Digestive Health, Division of Medicine, UCL, London, UK; 4 University College London (UCL) Centre for Advanced Biomedical Imaging, Division of Medicine, UCL, London, UK; stuart.taylor1@nhs.net

**Keywords:** Liver DCE MRI, liver perfusion, pharmacokinetic modelling

## Abstract

Delays between contrast agent (CA) arrival at the site of vascular input function (VIF) sampling and the tissue of interest affect dynamic contrast enhanced (DCE) MRI pharmacokinetic modelling. We investigate effects of altering VIF CA bolus arrival delays on liver DCE MRI perfusion parameters, propose an alternative approach to estimating delays and evaluate reproducibility.

Thirteen healthy volunteers (28.7  ±  1.9 years, seven males) underwent liver DCE MRI using dual-input single compartment modelling, with reproducibility (*n*  =  9) measured at 7 days. Effects of VIF CA bolus arrival delays were assessed for arterial and portal venous input functions. Delays were pre-estimated using linear regression, with restricted free modelling around the pre-estimated delay. Perfusion parameters and 7 days reproducibility were compared using this method, freely modelled delays and no delays using one-way ANOVA. Reproducibility was assessed using Bland–Altman analysis of agreement.

Maximum percent change relative to parameters obtained using zero delays, were  −31% for portal venous (PV) perfusion, +43% for total liver blood flow (TLBF), +3247% for hepatic arterial (HA) fraction, +150% for mean transit time and  −10% for distribution volume. Differences were demonstrated between the 3 methods for PV perfusion (*p*  =  0.0085) and HA fraction (*p*  <  0.0001), but not other parameters. Improved mean differences and Bland–Altman 95% Limits-of-Agreement for reproducibility of PV perfusion (9.3 ml/min/100 g, ±506.1 ml/min/100 g) and TLBF (43.8 ml/min/100 g, ±586.7 ml/min/100 g) were demonstrated using pre-estimated delays with constrained free modelling.

CA bolus arrival delays cause profound differences in liver DCE MRI quantification. Pre-estimation of delays with constrained free modelling improved 7 days reproducibility of perfusion parameters in volunteers.

## Introduction

Dynamic contrast enhanced (DCE) MRI is an established technique for quantification of liver perfusion. After the intravenous administration of gadolinium-based contrast agent (CA), images are acquired at high temporal resolution and dynamic changes in tissue signal intensity (SI) over time are recorded. SI measurements are then converted to CA concentration, before pharmacokinetic modelling of the uptake and washout of CA from the tissues is used to characterise tissue perfusion (Tofts and Kermode 1991, Materne *et al* 2002, Pandharipande *et al* 2005). These measurements have been used both in the assessment of microvascular changes in fibrosis/cirrhosis (Annet *et al* 2003, Hagiwara *et al* 2008, Kim *et al* 2008) and for assessment of lesional vascularity and tumour angiogenesis (Jackson *et al* 2002).

Liver DCE MRI is uniquely complex because of the dual portal venous (PV) and hepatic arterial (HA) blood supply. Pharmacokinetic modelling is reliant on simultaneous measurement of vascular input functions (VIFs) from regions-of-interest (ROIs) placed over afferent arterial and PV vessels. The modelling process convolves these functions with tissue enhancement curves to derive inflow and outflow constants. CA boluses arrival times differ between the arterial input function (AIF), PV input function (PVIF) and the liver parenchyma—to correct for this, terms for VIF-tissue bolus arrival delays are included in the model (Materne *et al* 2000, 2002, Miyazaki *et al* 2008).

Visual estimation of CA bolus arrival time is challenging because background noise can make appreciation of subtle SI changes difficult. Furthermore, limitations in the temporal resolution of the data acquisition can miss the exact CA bolus arrival time. Small changes in assumed AIF to tissue bolus arrival delays however cause major alterations in DCE computerised tomography (CT) estimated perfusion parameters, both within the liver (Miyazaki *et al* 2008) and other organs (Wu *et al* 2003). Additionally, VIF delays themselves are likely to be affected by the systemic and local haemodynamic changes induced by liver disease and would therefore be important to consider in the quantification process.

Various approaches to dealing with these delays are reported in the literature, including assuming zero delay (Murase *et al* 2007, Miyazaki *et al* 2008), fixing the delay across subjects for one or both VIFs (Materne *et al* 2002, Annet *et al* 2003) or free modelling of one or both of the VIF delays (Hagiwara *et al* 2008, Kim *et al* 2008). Each approach has limitations: free modelling for example estimates delays to optimise model fitting, but the addition of variables can result in non-physiological delay parameters (e.g. PVIF CA bolus arriving before the AIF) to achieve good ‘mathematical model fits’(Sourbron and Buckley 2012). An individualised approach that uses raw data to inform estimates of VIF delays whilst constraining them with physiologically acceptable limits could prove useful in deriving more physiological and accurate liver DCE MRI perfusion quantification.

In this study we investigate the effect of altering VIF CA bolus arrival delays on dual-input single compartment liver DCE MRI perfusion quantification in normal volunteers. Thereafter, we propose an alternative method to estimate AIF and PVIF CA bolus arrival delays and compare the 7 days reproducibility of derived perfusion measurements with alternative methods.

## Methods

### Subjects and preparation

Local ethics committee approval was obtained and participants provided informed written consent. Volunteers were recruited via advertisement within the university campus and were eligible if (a) they had no MRI contraindication, (b) were not taking any long-term medication (excluding the oral contraceptive pill) and (c) had no documented history of previous liver or gastrointestinal disease. Fourteen volunteers were screened of which one was excluded because of claustrophobia. The final cohort consisted of seven males (aged 26.5  ±  1.4 years) and six females (aged 31.2  ±  2.6 years). Participants fasted for 6 h prior to MRI and avoided caffeinated fluids. A 19G cannula was sited in a peripheral upper limb vein in preparation for administration of contrast. The breathing protocol was then explained to subjects before entering the scanner by the study coordinator (radiology research fellow with 5 years experience). For reproducibility studies, nine subjects consented to be re-scanned 7 days after the original study following identical preparation and MRI protocol, at a comparable time of the day (within 2 h).

### DCE MRI

Imaging was performed using a 3.0T scanner (Achieva, Philips Healthcare, Best, Netherlands) using a 16 channel body coil (SENSE XL-Torso, Philips Healthcare, Best, Netherlands). After initial anatomical imaging using a breath hold balanced steady-state free precession (SSFP) sequence, DCE studies were planned to ensure inclusion of the whole liver volume, retroperitoneal great vessels and the heart. A multi-flip angle T1 measurement was undertaken using three-dimensional (3D) gradient echo imaging at five different flip angles (5, 7, 10, 15 and 20°), with phase based B_1_ mapping for B_1_ non-uniformity correction (Treier *et al* 2007). DCE imaging in the coronal plane to minimise inflow effects, was performed using a 3D gradient turbo field echo (TFE) imaging with spectral attenuated inversion recovery (SPAIR) fat suppression. Thirty overcontiguous slices were acquired, then interpolated to sixty, with a total dynamic scan time of 3.35 s per 15 cm volume, scanned sequentially for 5 min (sequence parameters given in table 1). After the first five volumes were acquired, ten ml of Gd-DOTA (gadoterate dimeglumine, Dotarem^®^, Guerbet, Roissy, France), diluted in 10 ml of normal saline, was injected at 4 ml s^−1^ (Spectris^®^, Medrad Inc., USA), followed by a 20 ml saline flush. Subjects were given the first breath hold instruction before the CA injection: this minimised the likelihood of motion artefact in the early part of the DCE study (including the VIF peaks). Thereafter they were asked to continue self-directed breath holds in expiration for the duration of the study.

**Table 1.** Sequence parameters.

	T1 multi-flip angle	B1 map	dce mri (TFE with SPAIR fat suppression)
TR/TE (seconds)	4.0/2.0	100/1.0	2.3/1.0
Flip angle (°)	5, 7, 10, 15, 20	60	10
Matrix size (pixels)	240 × 240	100 × 100	240 × 240
Field-of-view (mm)	475 × 475	475 × 475	475 × 475
Spatial resolution (mm^2^)	1.98 × 1.98	4.75 × 4.75	1.98 × 1.98
Bandwidth (Hz/pixel)	389	1447	1411
Slice thickness (mm)	2.5	5	5
Slice gap (mm)	—	5	—
Slices per volume	60	30	30 (interpolated to 60)
SPAIR inversion time (ms)	—	—	56
Partial Fourier factor	—	—	0.625
TFE shots	—	—	16
TFE shot duration (ms)	—	—	209
Parallel imaging (SENSE) factors			2.9 RL, 1.4 AP

### Post-processing and data analysis

**Figure 1.**
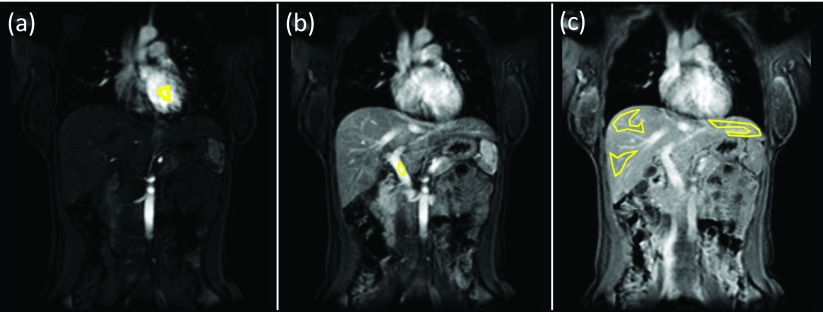
Example of ROI placement for DCE MRI quantification. Intra-ventricular ROI placement for AIF (a), PV ROI placement of PVIF (b) and parenchymal ROI placement (c) for segments II/III (far left), segments V/VI (right lower) and segments VII/VIII (right upper). Parenchymal ROIs were placed in each of the three locations on five slices.

Post-processing was performed using Matlab code (MathWorks, Natick, USA) developed in house. DCE volumes corrupted by significant motion artefact noise were discarded (average of 21/90 volumes discarded). No VIF peaks were missed in discarded data sets. Five coronal slices centred around the portal vein, each separated by 10 mm were then selected for analysis. Each slice was matched to data from the previously derived T1 maps and registered using robust data decomposition registration to correct for tissue displacement and deformation (Hamy *et al* 2014). Missing SI data from discarded volumes was estimated using linear interpolation. The interval for interpolation only exceeded one discarded volume on two occasions, both well after VIF and parenchymal peak SIs. In these instances, interpolation for two successive discarded volumes took place. Pixel wise conversion of sequential post-contrast SI into CA concentration was then undertaken using previously described methods for each of the five slices (Aronhime *et al* 2014, Gill *et al* 2014). Three parenchymal ROIs were positioned on each slice (total 15 ROIs), firstly in the right upper region (segments VII/VIII), left liver (segments II/III) and right lower region (segments V/VI). Care was taken to ensure parenchymal ROIs excluded any major inflow or outflow vessels (HA, PV and hepatic venous radicles). ROIs were then also positioned within the left ventricle and PV to derive each VIF (figure 1), as the left ventricle in our experience delivered more consistent VIFs. Perfusion parameters (detailed below) extracted from all fifteen ROIs (three ROIs on five slices) were averaged across all subjects for different post-processing method comparisons. All post-processing was undertaken by the study coordinator (radiology research fellow with 5 years’ experience of abdominal MRI).

### Pharmacokinetic modelling

**Figure 2.**
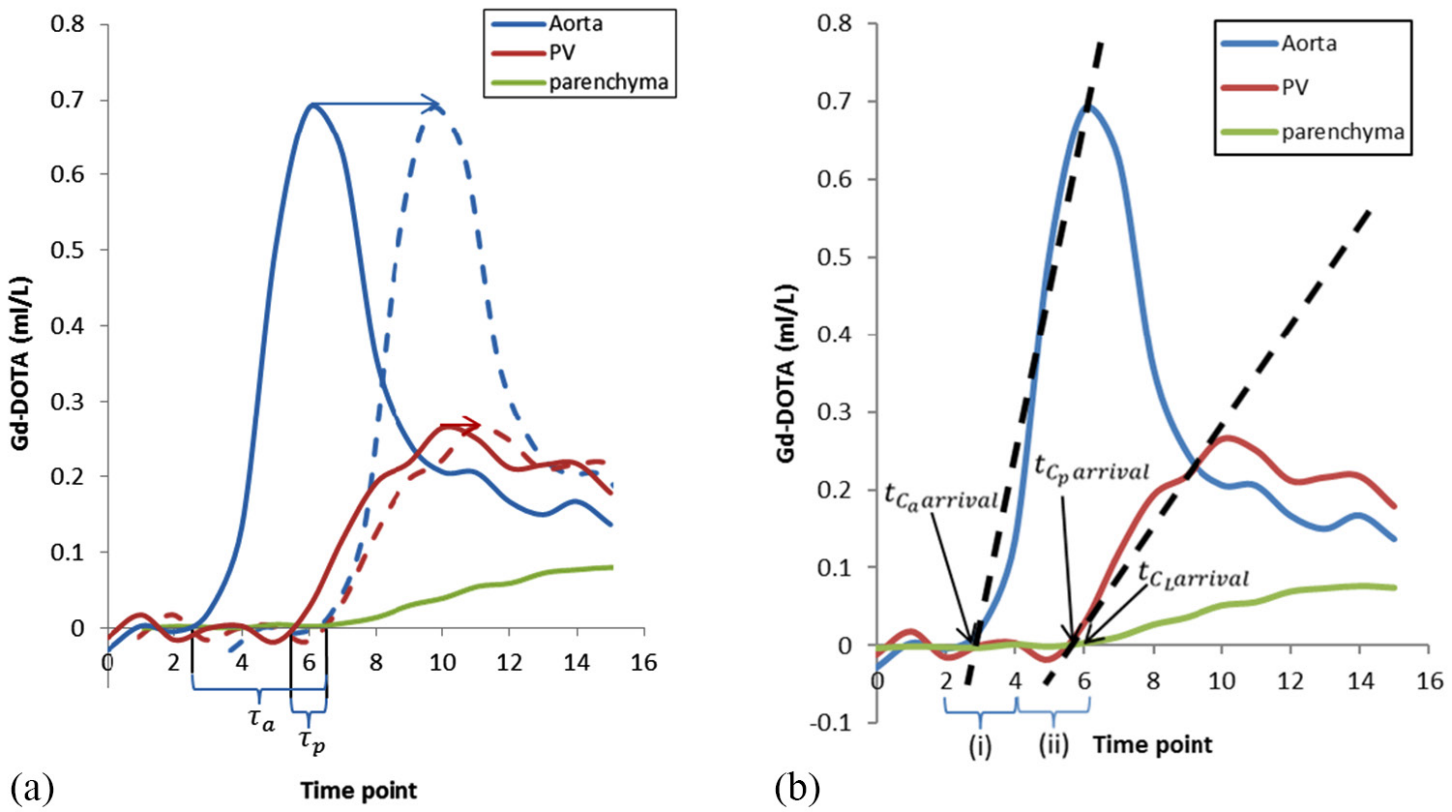
*τ*_a_, *τ*_p_, and linear regression for pre-estimation of AIF and PVIF CA bolus arrival delays. (a) CA bolus arrival delays between the afferent vessel and the liver are shown for the aorta (*τ*_a_) and PV (*τ*_p_). Inclusion of delay parameters has the effect of shifting the AIF (blue arrow and blue dashed AIF) and PVIF (red arrow and red dashed AIF) forward, to eliminate the delay to parenchymal enhancement (green enhancement curve) before fitting the data to the model. (b) Enhancement data between the first data point above the 95% confidence interval of the baseline and the VIF upstroke peak was modelled using linear regression to estimate aortic (${{t}_{{{C}_{\text{a}}}\text{arrival}}}$) and PV (${{t}_{{{C}_{\text{p}}}\text{arrival}}}$) CA bolus arrival times. Parenchymal CA arrival time (${{t}_{{{C}_{\text{L}}}\text{arrival}}}$) was determined using the 95% upper limit confidence interval of baseline data. Pharmacokinetic modelling was then undertaken constraining *τ*_a_ and *τ*_p_ to the limits specified by (i) and (ii) respectively.

Dual input single compartment modelling was undertaken as reported previously (Materne *et al* 2002, Hagiwara *et al* 2008). Briefly, liver parenchymal CA concentration as a function of time (${{C}_{\text{L}}}(t)$) can be expressed as:

1 \begin{eqnarray*}{{C}_{\text{L}}}(t)={\int}_{0}^{t}\left[{{k}_{1\text{a}}}{{C}_{\text{a}}}\left({{t}^{\prime}}-{{\tau}_{\text{a}}}\right)+{{k}_{1\text{p}}}{{C}_{\text{p}}}\left({{t}^{\prime}}-{{\tau}_{\text{p}}}\right)\right]{{\text{e}}^{-{{k}_{2}}\left(t-{{t}^{\prime}}\right)}} ~\text{d}{{t}^{\prime}}\,\end{eqnarray*}

where ${{C}_{\text{a}}}(t)$ represents the arterial input CA concentration as a function of time, ${{C}_{\text{p}}}(t)$ represents the PV input CA concentration as a function of time, ${{k}_{1\text{a}}}$ represents the arterial inflow constant, ${{k}_{1\text{p}}}$ represents the PV inflow constant, ${{k}_{2}}$ represents the outflow constant, ${{\tau}_{\text{a}}}$ represents the delay between the arrival of CA in the AIF and parenchymal ROIs and ${{\tau}_{\text{p}}}$ represents the delay between arrival of CA in the PVIF and parenchymal ROIs (figure 2(a)). Model fitting was undertaken using non-linear least squares fitting with in house developed Matlab code. Inflow and outflow constants were used to derive estimates of PV perfusion (ml/min/100 g), (TLBF, sum of HA and PV perfusion, ml/min/100 g), HA fraction (%), distribution volume (DV, %) and mean transit time (MTT, seconds) as reported previously (Materne *et al* 2002, Hagiwara *et al* 2008).

### Effects of altering VIF CA bolus arrival delays

CA bolus arrival delays in the modelling process effectively shift the VIFs in the modelling process forward by increments defined by the temporal resolution of the data (3.35 s in this study) (figure 2(a)). To investigate the effects of altering AIF and PVIF CA bolus arrival delays, pharmacokinetic modelling was undertaken after introducing successive increments to ${{\tau}_{\text{a}}}$ (6 steps, up to 20.10 s) and ${{\tau}_{\text{p}}}$ (3 steps, up to 10.05 s) for each dataset (larger delays were not studied as these are less likely to be physiological). Perfusion parameters for each combination of VIF delays (*n*  =  18) were then averaged across subjects for analysis.

### Alternative approaches to CA bolus arrival delays

Perfusion parameters and their 7 days reproducibility were then compared using the following methods for handling CA bolus arrival delays:

(i) No delays
  Assuming no delay between VIFs and parenchymal enhancement (i.e. ${{\tau}_{\text{a}}}$ and ${{\tau}_{p}}$ both set to zero), fixed across all datasets (Murase *et al* 2007, Miyazaki *et al* 2008).
(ii) Freely modelled delays
  Free (unconstrained) modelling of AIF and PVIF delays to optimise model fitting by minimising the residual sum of squares (Hagiwara *et al* 2008, Kim *et al* 2008).
(iii) Pre-estimated delays with constrained free modelling
  The first five data points for VIF and parenchymal CA concentrations were used to determine the baseline pre-CA concentration for each curve. Linear regression between the first VIF data point exceeding the upper limit of the 95% confidence interval of baseline pre-CA concentration, and the VIF peak was undertaken for the AIF and PVIF. The VIF CA bolus arrival times (${{t}_{{{C}_{\text{a}}}\text{arrival}}}$ and ${{t}_{{{C}_{\text{p}}}\text{arrival}}}$) were then each estimated as the point of intercept of the regression line with the time axis. Parenchymal enhancement was less noisy because of larger ROI size and lower susceptibility to flow artefact. The parenchymal CA arrival time (${{t}_{{{C}_{\text{L}}}\text{arrival}}}$) was then defined as the last data point before the parenchymal CA concentration exceeded the upper limit of the 95% confidence interval of the baseline pre-CA concentration. Figure 2(b) demonstrates the process in detail. Pre-estimates for ${{\tau}_{\text{a}}}$ and ${{\tau}_{\text{p}}}$ were then determined as:
  2 \begin{eqnarray*}\tau _{\text{a}}^{\prime}={{t}_{{{C}_{\text{L}}}\text{arrival}}}-{{t}_{{{C}_{\text{a}}}\text{arrival}}}\end{eqnarray*}
  3 \begin{eqnarray*}\tau _{\text{p}}^{\prime}={{t}_{{{C}_{\text{L}}}\text{arrival}}}-{{t}_{{{C}_{\text{p}}}\text{arrival}}}\end{eqnarray*}
  As $\tau _{\text{a}}^{\prime}$ and $\tau _{\text{p}}^{\prime}$ represented estimates of VIF delays, limited by temporal resolution (3.35 s), the pre-estimates were then used to constrain the range in which pharmacokinetic free modelling of ${{\tau}_{\text{a}}}$ and ${{\tau}_{\text{p}}}$ could occur, to one time point before and one time point after each estimate (i.e. within a 6.7 s window).

### Statistical analysis

To investigate the effect of altering AIF and PVIF CA bolus delays, calculated perfusion parameters for each delay were expressed as a percentage of those obtained when assuming zero delay between VIFs and parenchymal enhancement.

Kolmogorov–Smirnov tests were then used to confirm the normality of perfusion parameters derived (i) assuming no VIF delays (i.e. ${{\tau}_{\text{a}}}$ and ${{\tau}_{\text{p}}}$ both set to zero), (ii) freely modelled delays and (iii) pre-estimated delays with constrained free modelling. Repeated measures one-way analysis of variance (ANOVA) with corrections for non-sphericity were used to compare perfusion parameters using each of the three approaches to VIF delay estimation. Post hoc Tukey’s test was then applied where significant differences were identified. Where variables were found not to be normally distributed, the Kruskal–Wallis test was used followed by post hoc Dunn’s test if significant differences were identified. Paired *t*-tests/Wilcoxon matched-pairs signed rank tests as appropriate were used to compare VIF delay estimations obtained using freely modelled and pre-estimated delays with constrained free modelling. Seven-day reproducibility (*n*  =  9) was assessed using Bland–Altman (BA) analysis of agreement, with calculation of the mean difference (bias), 95% limits of agreement (LoA) and coefficients of variation. The threshold of statistical significance was defined to be *p*  <  0.05.

## Results

### Effects of altering VIF CA bolus arrival delays

**Figure 3.**
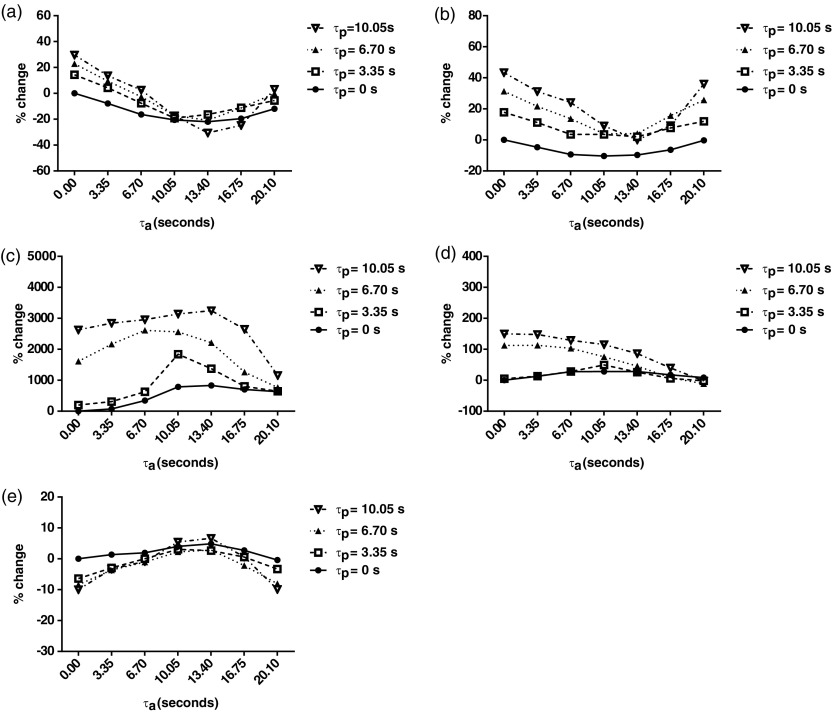
Effects of changes in CA bolus arrival delays on dual input single compartment parameter estimation. Percentage change relative to parameters calculated using zero CA bolus arrival delays are demonstrated for CA bolus arrival delays upto 20.10 s (*τ*_a_) and 10.05 s (*τ*_p_), for (a) PV perfusion, (b) TLBF, (c) HA fraction, (d) MTT and (e) DV.

Calculated perfusion parameters for each AIF and PVIF delay increment expressed as a percentage of those obtained when assuming zero delay (i.e. ${{\tau}_{\text{a}}}$ and ${{\tau}_{\text{p}}}$ set to zero) are shown in figure 3.

Estimated PV perfusion (figure 3(a)) decreased by as much as 31% (13.40 s AIF CA bolus arrival delay). Introducing PVIF CA bolus arrival delays increased PV perfusion by as much as 30% (10.05 s delay). A similar trend was demonstrated for TLBF (figure 3(b)), with perfusion estimates decreasing by as much as 10% (10.05 s AIF CA bolus arrival delay). Introducing PVIF CA bolus arrival delays increased TLBF by as much as 43% (10.05 s delay).

Because of small HA fraction estimates obtained when assuming zero VIF CA bolus arrival delays, introduction of CA bolus delays for both AIF and PVIFs resulted in increases of as much as 3247% (13.40 s AIF delay, 10.05 s PVIF delay, figure 3(c)). MTT increased by as much as 150% (0 s AIF delay, 10.05 s PVIF delay, figure 3(d)). DV reduced by up to 10% (0 s and 20.10 s AIF delay, 10.05 s PVIF delay, figure 3(e)).

### Comparison of VIF delay estimation methods

**Figure 4.**
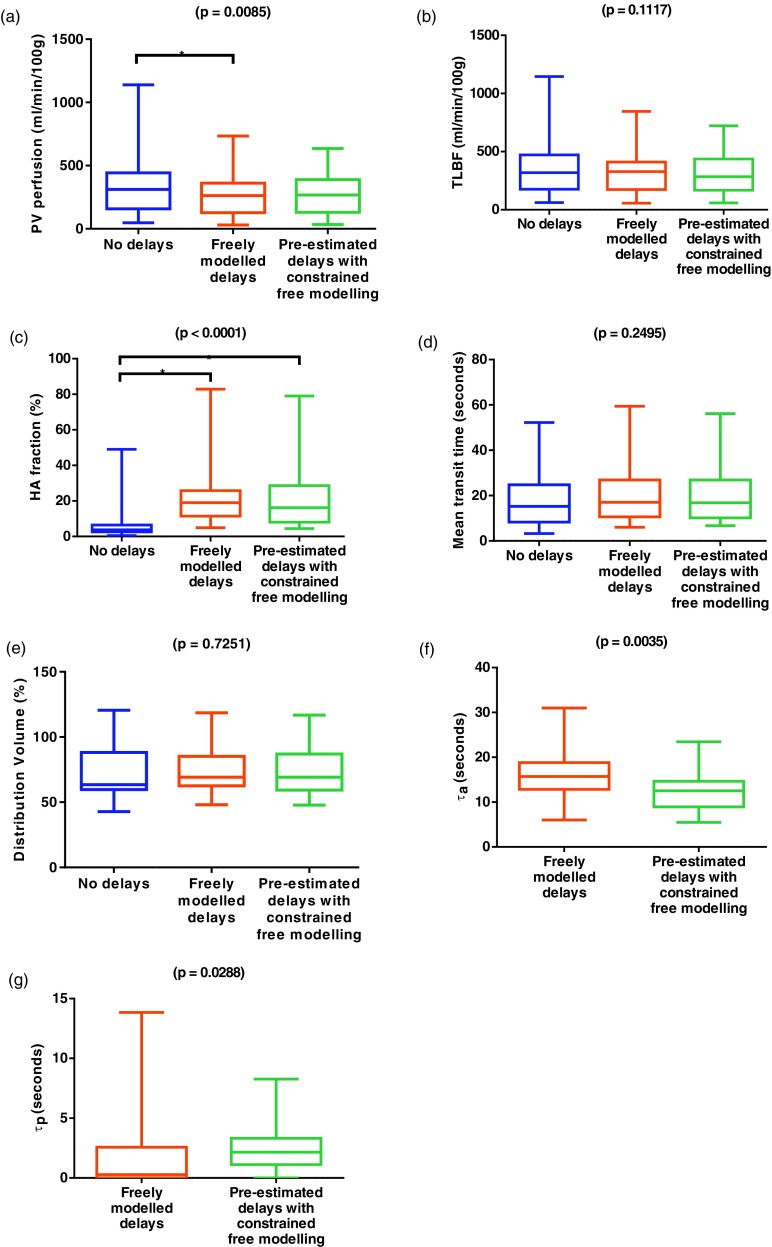
Perfusion parameters estimated using the dual input single compartment model, with each method of VIF delay estimation. *p*-values are quoted for one-way ANOVA/Kruskal–Wallis tests and paired *t*-tests/Wilcoxon matched-pairs signed rank tests where appropriate, with significant differences on post hoc testing (^*^), for no delays, freely modelled delays and pre-estimated delays with constrained free modelling ((a)–(e)). Comparisons of delay parameters were only undertaken when these were modelled (f) and (g).

Perfusion parameters calculated using each of the three methods are shown in table 2 and graphically in figure 4. HA fraction, DV and ‘*τ*_p_’ were not normally distributed and underwent non-parametric statistical testing. Significant differences were demonstrated between the three methods for PV perfusion (*F*(1.25, 24.96)  =  7.29; *p*  =  0.0085) and HA fraction (*H*  =  23.94; *p*  <  0.0001), but there were no significant differences for the other parameters. Post hoc testing demonstrated significant differences between zero CA bolus VIF arrival delays and freely modelled delays for both PV perfusion and HA fraction, but only for HA fraction when free modelling was constrained using pre-estimated delays. Significant differences were demonstrated between freely modelled and pre-estimated delays with constrained free modelling for AIF (mean difference  −3.7  ±  1.1 s, *p*  =  0.0035) and PVIF CA bolus arrival delays (median difference 1.12 s, *p*  =  0.029).

**Table 2.** Perfusion parameters estimated using the dual input single compartment model, with each method of VIF delay estimation.

	No delays	Freely modelled delays	Pre-estimated delays with constrained free modelling
PV perfusion (ml/min/100 g)^a^	351.9 ± 55.1	262.7 ± 37.4^b^	274.3 ± 38.4
TLBF (ml/min/100 g)	367.3 ± 54.6	321.0 ± 41.2	327.5 ± 41.7
HA fraction (%)^a^	7.4 ± 2.3	21.7 ± 3.6^b^	20.7 ± 3.7^b^
Mean transit time (seconds)	18.6 ± 2.7	20.0 ± 2.7	19.9 ± 2.6
Distribution volume (%)	71.2 ± 4.3	74.1 ± 3.9	73.5 ± 4.0
*τ*_a_ (seconds)^a^	—	16.1 ± 1.3	12.5 ± 1.1
*τ*_p_ (seconds)^a^	—	1.9 ± 0.7	2.6 ± 0.5

a One-way ANOVA/Kruskal–Wallis/paired *t*-test/Wilcoxon rank *p*  <  0.05.

b Post hoc Tukey test comparison with no delays *p*  <  0.05.

### Reproducibility studies

**Figure 5.**
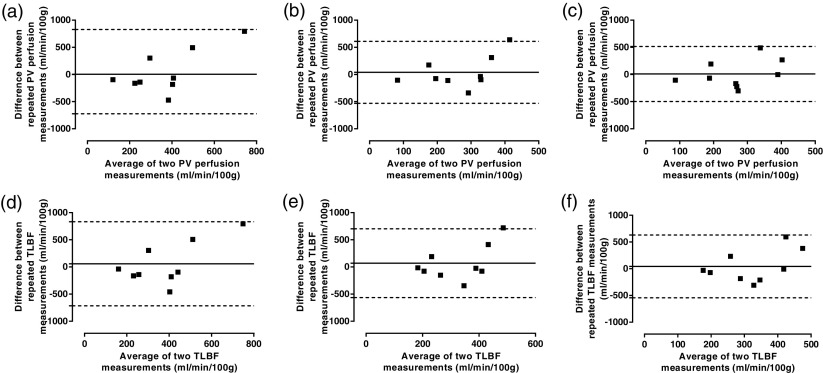
Analysis of agreement of absolute perfusion parameters using alternative approaches to VIF delays with dual input single compartment modelling. Bland–Altman analysis of PV perfusion (upper row) and TLBF (lower row) using ((a) and (d)) zero VIF delays, ((b) and (e)) free modelling of VIF delays and ((c) and (f)) constrained free modelling of pre-estimated VIF delays.

**Figure 6.**
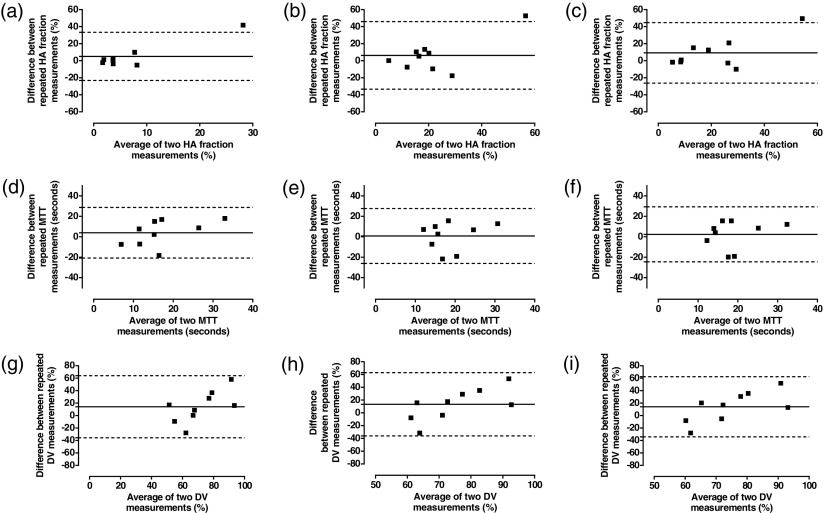
Analysis of agreement of relative perfusion parameters using alternative approaches to VIF delays with dual input single compartment modelling. Bland–Altman analysis of HA fraction (upper row), MTT (middle row) and DV (lower row) using ((a), (d) and (g)) zero VIF delays, ((b), (e) and (h)) free modelling of VIF delays and ((c), (f) and (i)) constrained free modelling of pre-estimated VIF delays.

Reproducibility was assessed in 9 normal volunteers 7 days after the initial study (table 3, figures 5 and 6). The mean difference and BA 95% LoAs for repeated PV perfusion and TLBF measurements were smallest using pre-estimated delays with constrained free modelling. The coefficient of variation using this method was similar to unconstrained free modelling for both parameters.

**Table 3.** Summary of reproducibility of perfusion parameters estimated using alternative approaches to VIF delays with dual input single compartment modelling alongside PCMRI reproducibility.

	No delays	Freely modelled delays	Pre-estimated delays with constrained free modelling
**PV perfusion (ml/min/100 g)**			
Mean difference	52.9	40.1	**9.3**
BA 95% LoA	±776.6	±570.6	**±506.1**
Coefficient of variation	71.8%	65.2%	**64.1%**

**TLBF (ml/min/100 g)**			
Mean difference	58.6	69.6	**43.8**
BA 95% LoA	±773.8	±633.6	**±586.7**
Coefficient of variation	68.2%	58.9%	**58.3%**

**HA fraction (%)**			
Mean difference	**5.1**	6.3	9.3
BA 95% LoA	**±28.3**	±39.6	±35.5
Coefficient of variation	145.3%	**76.4%**	81.7%

**Mean transit time (seconds)**			
Mean difference	4.0	**0.8**	2.4
BA 95% LoA	**±24.8**	±26.8	±26.9
Coefficient of variation	65.6%	61.3%	**60.8%**

**Distribution volume (%)**			
Mean difference	14.2	**13.4**	14.1
BA 95% LoA	±49.9	±49.5	**±48.2**
Coefficient of variation	27.8%	**23.8%**	24.7%

*Note*: Emboldened values in the table highlight the best performing method for each statistic.

The mean difference and BA 95% LoAs between repeated HA fraction measurements was smallest using no delays. The coefficient of variation was smallest using freely modelled delays. The smallest mean difference for repeated MTT and DV measurements was demonstrated using freely modelled delays. BA 95% LoAs and coefficients of variation were similar across all three methods for both MTT and DV (table 3).

## Discussion

We have investigated the effects of CA bolus arrival delays on pharmacokinetic parameter estimation using non-simulated human liver DCE MRI data. The perfusion parameter variation driven by changes in CA bolus arrival delay parameters underlines the importance of proper consideration of delays in the quantification process. A robust method to measure these accurately and consistently across thousands of pixels, each with physiological (as a result of distance from VIF ROIs) and pathological variations (as in the case of focal lesions or heterogeneous diffuse liver disease) in CA bolus arrival delay is essential.

Based on our data, we would propose pre-estimation of delays with constrained free modelling as a useful strategy. Whilst the coefficients of variation of freely modelled delays and pre-estimated delays with constrained free modelling are similar across all perfusion parameters, the improved reproducibility of absolute perfusion parameters (i.e. PV perfusion and TLBF), as demonstrated by BA 95% LoAs is a major strength. These are the most conceptually useful clinical parameters with potential as vascular biomarkers of liver function (Chouhan *et al* 2016). Such improvements, especially when arising from more physiological estimates of bolus arrival delay represent a clinically useful development relative to the use of freely modelling.

The reproducibility of HA fraction using our proposed method is disappointing but is likely related to underestimation of HA fraction when using no CA bolus arrival delays. Similar reproducibility across the three methods for MTT and DV is likely accounted for by their reliance on the outflow constant (${{k}_{2}}$). The latter is less affected by CA bolus arrival time and more reliant on the tail portions of enhancement curves. The clinical value of MTT and DV in the context of liver pathology are not fully understood but changes in PV perfusion and TLBF would have potential use in the vascular assessment of liver disease (e.g. measurement of the HA buffer response (Lautt 2007) or critical hypoperfusion in liver failure (Mehta *et al* 2014)), or in the assessment of lesional vascularity and tumour angiogenesis (Annet *et al* 2003, Abdullah *et al* 2008, Hagiwara *et al* 2008, Patel *et al* 2010, Ferl and Port 2012, Cao *et al* 2013).

Although a strength of our study is the use of prospectively acquired human DCE data, there are limitations. We had no standard of reference for liver perfusion parameters and therefore cannot determine which method is most accurate. To overcome this, we compared methods using 7 days reproducibility. We also acknowledge that by expressing the effects of changes in CA bolus arrival delays using percent change, the effect small absolute increments in low absolute values can be exaggerated (as in the case of HA fraction). It is also of note that changes in TLBF are expectably similar to those recorded for PV perfusion, as TLBF is predominantly composed of PV perfusion. Finally there is limited published data on the reproducibility of liver DCE MRI using dual-input single compartment modelling, but our data demonstrates relatively wide BA 95% LoAs and coefficients of variation for perfusion parameters. Aronhime *et al* for example, reported comparably wide coefficients of variation of 58%, 39%, 73% and 15% for PV perfusion, TLBF, HA fraction and DV respectively (Aronhime *et al* 2014). While we acknowledge that weak reproducibility does undermine the clinical utility of liver DCE MRI, we believe this may reflect natural variation in perfusion, contingent on differences in subject hydration, but also the many other challenges in performing clinical DCE MRI not directly addressed by the present study.

Several methodological details have the potential to significantly affect quantification. All delay estimations (for freely modelled and pre-estimated delays with constrained free modelling) are restricted to unit shifts defined by the temporal resolution of the data (in this study, 3.35 s). Accurate T1 measurements for example, are essential. Our use of multi-flip angle T1 measurements with phase-based B_1_ mapping/B_1_ non-uniformity correction is not without error, but based on prior experience deemed suitable for this application (Barnes *et al* 2014). Post-processing is also heavily reliant on complex computation and intensive human input. Discarding on average 21/90 volumes though not unexpected (assuming an average breath hold of 15 s, equating to 20 inspiration/expiration cycles over 5 min) could also affect quantification. The use of linear interpolation, motion correction, slice selection for analysis and ROI positioning also have the potential to affect results.

## Conclusion

We have shown that differences in CA bolus arrival delays can cause profound differences in dual-input single compartment modelled hepatic perfusion parameters. Such variations are a major barrier to cross-institution large-scale studies required to determine the clinical value of liver DCE MRI, and develop it as a universal tool. As a solution, we propose a simple method for estimation of AIF and PVIF CA bolus arrival delays to optimise model fitting within physiologically viable estimates of delays. We have demonstrated that this method produces similar perfusion parameter estimates to freely modelled CA bolus arrival delays, improves the reproducibility of PV perfusion and TLBF and provides comparable reproducibility to freely modelled delays for HA fraction, MTT and DV.
